# Hierarchical image classification using transfer learning to improve deep learning model performance for amazon parrots

**DOI:** 10.1038/s41598-025-88103-3

**Published:** 2025-01-30

**Authors:** Jung-Il Kim, Jong-Won Baek, Chang-Bae Kim

**Affiliations:** Biotechnology Major, Sangmyung University, Seoul, 03016 South Korea

**Keywords:** Genus *Amazona*, CITES, Conservation, Object detection, Hierarchical transfer classification, Biodiversity, Conservation biology, Machine learning

## Abstract

Numerous studies have proven the potential of deep learning models for classifying wildlife. Such models can reduce the workload of experts by automating species classification to monitor wild populations and global trade. Although deep learning models typically perform better with more input data, the available wildlife data are ordinarily limited, specifically for rare or endangered species. Recently, citizen science programs have helped accumulate valuable wildlife data, but such data is still not enough to achieve the best performance of deep learning models compared to benchmark datasets. Recent studies have applied the hierarchical classification of a given wildlife dataset to improve model performance and classification accuracy. This study applied hierarchical classification by transfer learning for classifying Amazon parrot species. Specifically, a hierarchy was built based on diagnostic morphological features. Upon evaluating model performance, the hierarchical model outperformed the non-hierarchical model in detecting and classifying Amazon parrots. Notably, the hierarchical model achieved the mean Average Precision (mAP) of 0.944, surpassing the mAP of 0.908 achieved by the non-hierarchical model. Moreover, the hierarchical model improved classification accuracy between morphologically similar species. The outcomes of this study may facilitate the monitoring of wild populations and the global trade of Amazon parrots for conservation purposes.

**Supplementary Information:**

The online version contains supplementary material available at 10.1038/s41598-025-88103-3.

## Introduction

In the last few years, various studies have proven the potential of deep learning models as tools for classifying images for wildlife conservation^1–3^. Deep learning models have been successfully used to monitor wildlife using images obtained by the camera trap method^4,5^. Although this method has been widely and successfully applied for the monitoring of wildlife populations^1,6^, it can be challenging due to the need for manual analysis of a large number of images to identify species morphologically^7,8^. In this regard, deep learning models can substantially reduce the workload of experts by enabling the automatic classification of images following criteria set by experts, for example, classifying images by species and selecting images of specific species^1,6^. Furthermore, in previous studies deep learning models were successfully implemented for the classification of globally traded and frequently illegally traded wildlife species^2,9^. Identifying the traded wildlife is an essential first step in managing the global wildlife trade^10^. This is conventionally achieved by morphological identification of the species by experts^11,12^, but the increasing volume of such trade is overwhelming and difficult to track^13,14^. The initial classification of traded wildlife by deep learning models can help experts identify a vast amount of wildlife^13^. Among the deep learning models developed for image classification, convolutional neural networks (CNNs) are basic and standard models. Based on CNNs, object detection models were developed to detect and classify objects in images. Accurate wildlife detection is as crucial as classification in monitoring the distribution^15,16^, density^17^, and populations^18^ of wildlife to facilitate the conservation of vulnerable species. The You Only Look Once (YOLO) series models are currently at the forefront of object detection models^19–21^. Among these models, YOLO version 5 (YOLOv5) has been shown to surpass most object detection models in terms of accuracy and speed on Common Object in Context (COCO) datasets, which are large-scale benchmark datasets containing a variety of objects^21^. It has also been extensively used for classifying different organisms^22–24^.

In deep learning, model performance is generally improved by providing more extensive input data^25^. However, the amount of available image data of wildlife is typically a factor that limits robust learning, particularly for rare or endangered species^26^. Recently, citizen science programs involving volunteers with various levels of expertise have facilitated the accumulation of valuable wildlife data across spatial and temporal scales that are challenging to collect by conventional methods^27^. Although these programs provide an abundance of wildlife image data, such data are still inadequate to achieve the best performance of state-of-the-art deep learning models compared with benchmark datasets, such as COCO and ImageNet^28^. Accurate classification of species is vital for conservation efforts, as “species” is a widely accepted standard unit used in wildlife management to protect biodiversity from extinction and illegal trade^29^. However, when deep learning models are trained with insufficient image data, this can result in misclassification between morphologically similar species^2,9^. Thus, there is a need for a novel method to enhance the performance of deep learning models in accurately classifying wildlife using a given insufficient dataset.

Recent studies have suggested the value of hierarchically classifying a given dataset to improve model performance^30,31^. Hierarchical classification involves constructing local classification models that are organized according to a predefined hierarchical structure^32^. Hierarchical classification is conducted successively from higher to lower levels using a dataset comprising classes defined by the number of different labeled categories. Classes at lower levels are nested within classes at higher levels in the hierarchy, and classes at lower levels are referred to as children of the associated parent class at a higher level. Moreover, hierarchical classification has been applied by using a transfer learning concept, which transfers the knowledge from the pre-trained model using benchmark datasets to the new task model^33^. Hierarchical classification using transfer learning involves the transfer of knowledge from the pre-trained model with a parent dataset rather than a benchmark dataset to the model with a children dataset for training. It has been reported that this method achieved good classification performance in various fields, such as on medical images^30^ and fine-grained natural images^31^. Additionally, recent studies applied hierarchical classification on images of wildlife, which improved the classification accuracy^28,34–36^.

Among various types of wildlife, parrots (order Psittaciformes) are one of the most threatened groups due to the loss of their habitat^37^ and pet trade^38^. Conserving parrots is crucial, as they play a vital role in ecosystems by consuming and dispersing the reproductive structures of plants^39^. Parrots contribute to seed dispersal through external transport mechanisms involving their beaks and feet, as well as internal transport via ingestion and excretion^39^. In addition, they play a role in plant pollination and protection by consuming plant-based parasites^40^. Parrots from the family Psittacidae are among the most commonly traded bird species^10^. Within this family, Amazon parrots (genus *Amazona*), the most diverse parrot group, including 35 species, are among the most traded parrots^41^. On average, approximately 12,000 parrots from the Amazon region are exported annually to different countries, with the orange-winged Amazon parrot (*Amazona amazonica*) being the most frequently exported species^42^. According to the IUCN Red List of Threatened Species, four Amazon parrot species are categorized as “Critically Endangered,” five as “Endangered,” and eight as “Vulnerable”^43^. Additionally, the wild populations of 27 wild Amazon parrot species are currently shrinking^43^. Moreover, 16 Amazon parrot species are included in CITES Appendix I, indicating the strict prohibition of their trade^44^. To conserve these parrot species, a previous study applied a deep learning model to classify images of 26 Amazon parrot species^2^. That study indicated that the accuracy of classification between morphologically similar species was relatively low. Indeed, the classification accuracy of *Amazona vittata* was the lowest at 75.0%, primarily due to the 16.7% misclassification rate with the morphologically similar species *Amazona tucumana*. Amazon parrots are distinguished by their predominantly green bodies, with varying colors—primarily red, yellow, white, and blue—appearing on the head, breast, shoulders, and flight feathers^45–47^. These similar patterns can lead to misidentification of species^46,47^. Thus, to accurately and rapidly monitor wild parrot populations and regulate their global trade, it is essential to improve the accuracy of classification between morphologically similar Amazon. Achieving this would support conservation efforts and help prevent the illegal trade of these vulnerable species.

In the present study, we developed a hierarchical image classification model for Amazon parrots using transfer learning. The main objectives of this study were as follows: (1) to construct the hierarchy based on the diagnostic morphological features of the Amazon parrot species, (2) to develop the hierarchical and non-hierarchical models for detecting and classifying Amazon parrots, (3) to compare the overall performance of the hierarchical model to that of the non-hierarchical model to assess the capacity of the hierarchical approach to improve the classification of Amazon parrots of the hierarchical approach.

## Methods

### Collection of images

**Fig. 1:**
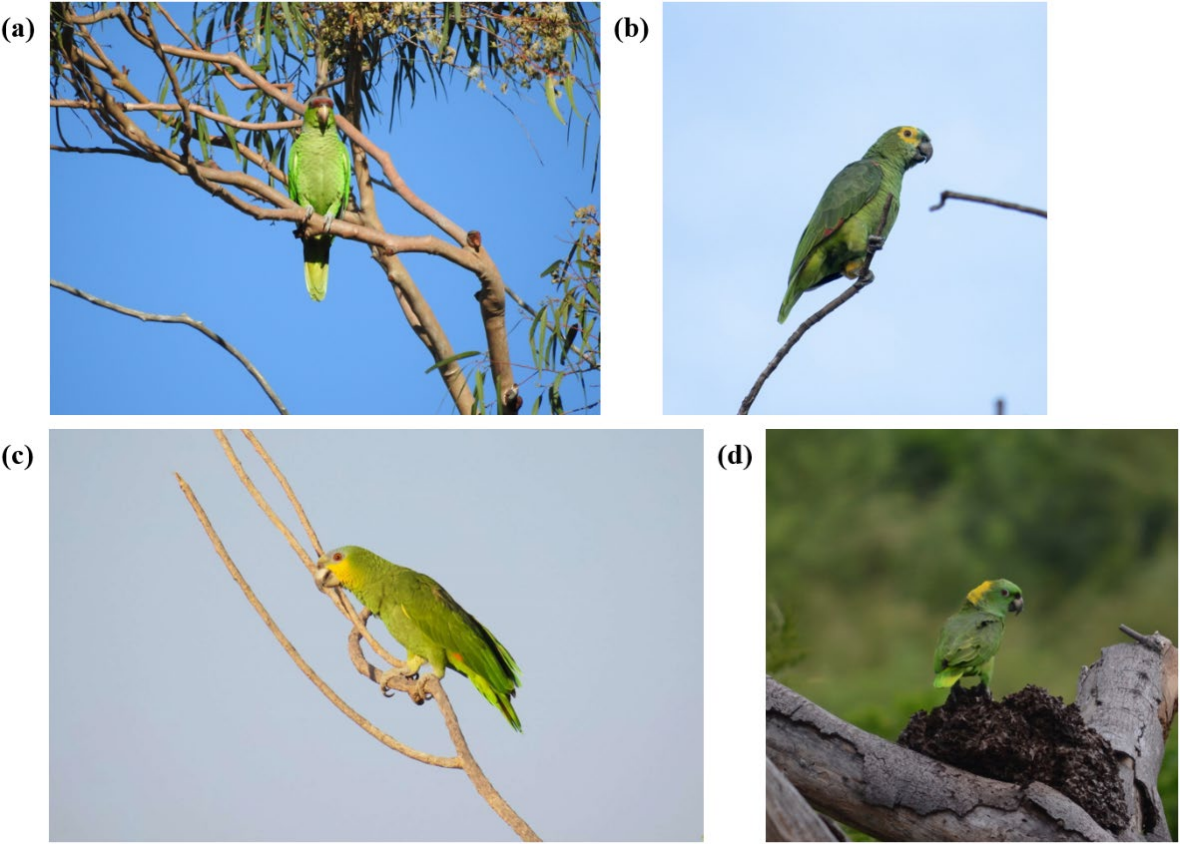
Representative images of examined species. (a) *Amazona finschi*, (b) *Amazona aestiva*, (c) *Amazona amazonica*, and (d) *Amazona auropalliata*. Photo credit: (a) Whitney Tsai Nakashima, (b) André M. Rodeguero Stefanuto, (c) Arman Moreno, and (d) Hans Holbrook.

The images of 35 Amazon parrot species were mainly obtained from eBird (www.ebird.org). To minimize data imbalance, images of species, for which there were fewer than 1,000 images in eBird were also gathered from iNaturalist (www.inaturalist.org) and Google Images (www.images.google.com). The iNaturalist images of research grade were collected using the Inat_images R script package^48^, and Google Images queries were performed using the scientific and common names of the parrots with a Python script^49^. Identification of species in the examined images was based on species-specific morphological features described in three expert books^45–47^. Any images for which accurate identification of the species could not be achieved were removed. Moreover, only images with a resolution of at least 500 × 500 pixels and minimum quality of 72 dpi were selected from the three databases. For species with gender dimorphism, such as *Amazona albifrons* and *Amazona viridigenalis*, images of both males and females were included^45–47^. Figure 1 presents representative images of examined species. The dataset included images taken from various angles, such as frontal (Fig. 1a), lateral (Fig. 1b and c), and dorsal (Fig. 1d), indicating the morphological features of Amazon parrot species, such as the crown, forehead, scapulars, and tail feathers. The number of images from each database is detailed in Table S1. As morphological features that can be used to identify species are distributed across the body, the entire body was annotated as a ground truth bounding box using LabelImg^50^.

## Construction of the hierarchy based on diagnostic morphological features

**Fig. 2:**
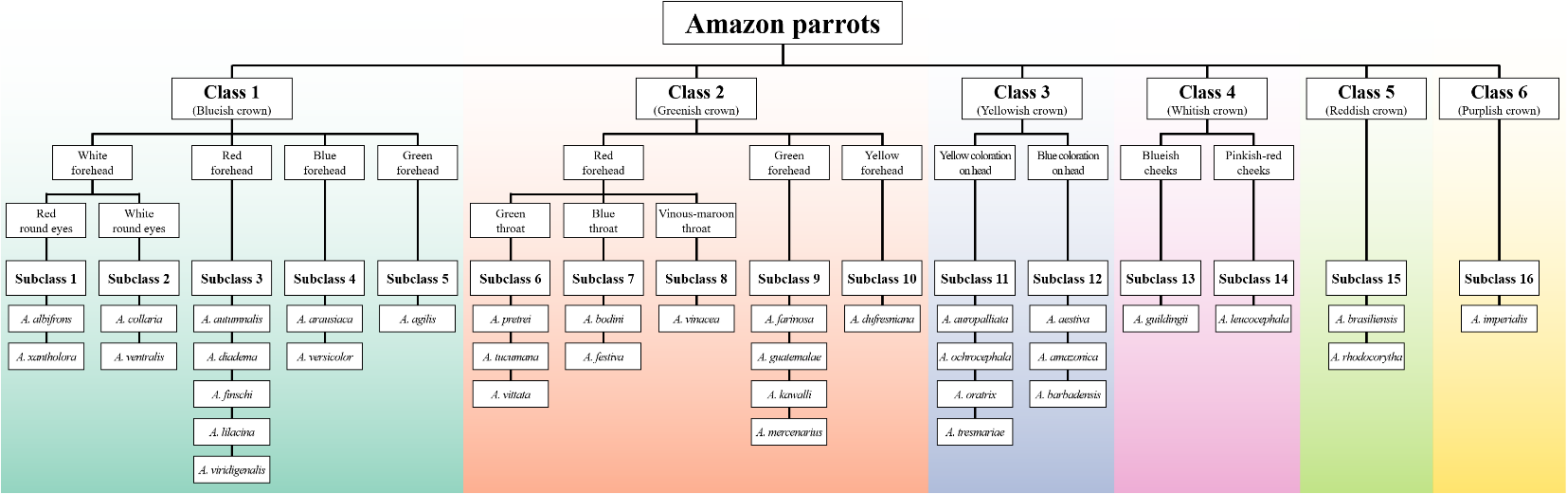
Hierarchy constructed based on the diagnostic morphological features of Amazon parrots.

The hierarchy constructed based on the diagnostic morphological features of the Amazon parrots is presented in Fig. 2 and Table S2. The diagnostic morphological features were selected with reference to three expert books^45–47^. Thirty-five Amazon parrot species were initially grouped into classes based on the crown color, which was set as the primary diagnostic morphological feature. Classes 1–6 consisted of species with blueish, greenish, yellowish, whitish, reddish, and purplish crowns, respectively. Then, each class was separated into subclasses based on secondary or tertiary diagnostic morphological features, such as forehead color, coloration on the head, cheek color, color around the eyes, and throat color. Most subclasses were grouped based on secondary diagnostic morphological features, forehead color, coloration on the head, and cheek color. Subclasses 1 and 2 shared primary and secondary diagnostic morphological features, but were separated by the tertiary feature of color around the eyes. Similarly, subclasses 6–8 shared primary and secondary diagnostic morphological features, but were separated by the tertiary feature of throat color. Subclasses 15 and 16 were the only ones included in classes 5 and 6, respectively, because the species in these classes possess distinctive primary morphological features, namely, a reddish or purplish crown, respectively. This made further subclassification unnecessary. Finally, 35 Amazon parrot species were grouped into six classes, including 16 subclasses, based on the diagnostic morphological features.

## Training of deep learning models

Third-level classifications, class, subclass, and species were conducted as part of the hierarchical classification process for Amazon parrots. This multi-level approach aimed to improve the ability of the model to distinguish between species by progressively narrowing down the classifications based on shared morphological features. To implement this hierarchical classification approach, the concept of transfer learning was employed to transfer knowledge from one level of classification to the next. This allows the model to build on the information learned at each stage, enhancing its accuracy and performance in the subsequent levels of classification. Specifically, a class-level classification model was first developed, using the dataset described in Table S3. Next, the subclass-level classification model was trained by leveraging knowledge transferred from the class-level classification model, using the dataset described in Table S4. Finally, the species-level model, referred to as the hierarchical model, was trained by leveraging the knowledge transferred from the subclass-level classification model using the dataset described in Table S5. Additionally, the non-hierarchical model was developed to compare its performance with that of the hierarchical model. The non-hierarchical model was trained without using transfer learning from the class- and subclass-level classification models, using the dataset described in Table S5. This comparison allowed for a direct evaluation of the effectiveness of the hierarchical approach. All datasets used to train the models consisted of training (64%), validation (16%), and test (20%) sets (Tables S3-5), following the widely accepted 8:2 dataset division ratio^28,51,52^. The hierarchical model was developed using the YOLOv5s, the standard structure of the YOLOv5 models^21^. For the training of models, images were unified as 640 × 640 pixels, and the batch size and epoch were set to 16 and 300, respectively. Furthermore, to prevent overfitting, two data augmentation techniques, namely, albumentation^53^ and mosaic augmentation^54^, were applied to the training dataset. The experimental setup was conducted on an Ubuntu 20.04 operating system, using two Intel Xeon Silver 4110 CPUs, RTX 2080 Ti graphics with 11GB of video memory, and four 16GB REG.ECC DDR4 SDRAMs. The experimental program was implemented with Python 3.11.5, PyTorch 2.1.0, and CUDA 12.1.

## Evaluation of model performance

In this study, various evaluation indexes were used to assess the model performance^21^. The loss function, a fundamental element in deep learning, measures the discrepancy between predicted outcomes and actual values. By quantifying the loss, the model receives feedback that allows it to optimize its performance and improve during training. Three specific loss functions were evaluated during the training process by using the validation set: Complete Intersection over Union (CIoU), classes, and objectness losses. The “CIoU loss” measures the mean value of the CIoU loss function, where the value is inversely proportional to the accuracy of the prediction box^55^. A lower “CIoU loss” indicates better alignment between the predicted bounding box generated by the model and actual bounding box labeled by the researcher. The “classes loss” refers to the average loss in the classification task, with its value being inversely proportional to the classification accuracy^55^. Finally, the “objectness loss” indicates the mean loss of the target detection confidence, where a lower value suggests higher confidence in detecting the target^55^. Both “classes and objectness losses” were computed using cross-entropy loss, while “CIoU loss” was calculated based on the Intersection over Union (IoU)^55^. The IoU, defined in formula (1), uses G and P to denote the ground truth and predicted bounding boxes, respectively. The “CIoU loss” was computed using formula (2), where denotes the distance between the center points of the two bounding boxes, and represents the diagonal length of the smallest enclosing box that encompasses both bounding boxes. The aspect ratio alignment between the two bounding boxes is represented by the term *v*, calculated using formula (3), where *w*^*gt*^ and *h*^*gt*^ ​denote the width and height of the ground truth bounding box, and *w* and *h* denote the width and height of the predicted bounding box, respectively. Finally, the trade-off parameter *α* was determined using formula (4), balancing the different components of the CIoU loss.

1 $$\:\text{I}\text{o}\text{U}=\frac{G\cap\:P}{G\cup\:P}\:$$

2 $$\:\text{C}\text{I}\text{o}\text{U}\:\text{l}\text{o}\text{s}\text{s}=1-\text{I}\text{o}\text{U}+\frac{{d}^{2}}{{c}^{2}}+\alpha\:v$$

3 $$\:v=\frac{4}{{\pi\:}^{2}}{(\text{a}\text{r}\text{c}\text{t}\text{a}\text{n}\frac{{w}^{gt}}{{h}^{gt}}-\text{a}\text{r}\text{c}\text{t}\text{a}\text{n}\frac{w}{h})}^{2}$$

4 $$\:\alpha\:=\frac{v}{\left(1-IoU\right)+v}$$

Precision, recall, and mean Average Precision (mAP) were used to evaluate model performance^21^. These metrics were assessed during training, using the validation set, and after training by using the test set. Precision is defined as the proportion of true results among the predicted results by the model, and recall measures the proportion of correctly predicted instances relative to the total actual instances. mAP reflects precision and recall, providing a comprehensive measure of model performance. Precision and recall were computed using the formulas (5) and (6), respectively. AP was calculated using formula (7), where *n* denotes the number of ground truth objects. AP provides a balance between precision and recall by integrating the area under the precision–recall curve, thereby optimizing detection and classification tasks. mAP was then derived using formula (8), where *Q* is the total number of queries in the dataset, and *AP(q)* represents the AP for each query *q*. In this study, mAP0.5 and mAP0.5–0.95 were considered, with mAP0.5 corresponding to an IoU threshold of 0.5, and mAP0.5–0.95 covering a range of IoU thresholds of 0.5–0.95. Additionally, the model’s classification results were visualized using a confusion matrix.

5 $$\:\text{P}\text{r}\text{e}\text{c}\text{i}\text{s}\text{i}\text{o}\text{n}=\frac{\text{T}\text{r}\text{u}\text{e}\:\text{P}\text{o}\text{s}\text{i}\text{t}\text{i}\text{v}\text{e}}{\text{T}\text{r}\text{u}\text{e}\:\text{P}\text{o}\text{s}\text{i}\text{t}\text{i}\text{v}\text{e}+\text{F}\text{a}\text{l}\text{s}\text{e}\:\text{P}\text{o}\text{s}\text{i}\text{t}\text{i}\text{v}\text{e}}$$

6 $$\:\text{R}\text{e}\text{c}\text{a}\text{l}\text{l}=\frac{\text{T}\text{r}\text{u}\text{e}\:\text{P}\text{o}\text{s}\text{i}\text{t}\text{i}\text{v}\text{e}}{\text{T}\text{r}\text{u}\text{e}\:\text{P}\text{o}\text{s}\text{i}\text{t}\text{i}\text{v}\text{e}+\text{F}\text{a}\text{l}\text{s}\text{e}\:\text{N}\text{e}\text{g}\text{a}\text{t}\text{i}\text{v}\text{e}}$$

7 $$\:\text{A}\text{v}\text{e}\text{r}\text{a}\text{g}\text{e}\:\text{P}\text{r}\text{e}\text{c}\text{i}\text{s}\text{i}\text{o}\text{n}\:\left(\text{A}\text{P}\right)=\:\sum\:_{x=0}^{x=n-1}\{\text{R}\text{e}\text{c}\text{a}\text{l}\text{l}\left(x\right)-\text{R}\text{e}\text{c}\text{a}\text{l}\text{l}\left(x+1\right)\}\times\:\text{P}\text{r}\text{e}\text{c}\text{i}\text{s}\text{i}\text{o}\text{n}\left(x\right)$$

8 $$\:\text{m}\text{e}\text{a}\text{n}\:\text{A}\text{v}\text{e}\text{r}\text{a}\text{g}\text{e}\:\text{P}\text{r}\text{e}\text{c}\text{i}\text{s}\text{i}\text{o}\text{n}\:\left(\text{m}\text{A}\text{P}\right)=\frac{\sum\:_{q=1}^{Q}\text{A}\text{P}\left(q\right)}{Q}$$

## Results

### Comparison of performance during the training process

**Fig. 3:**
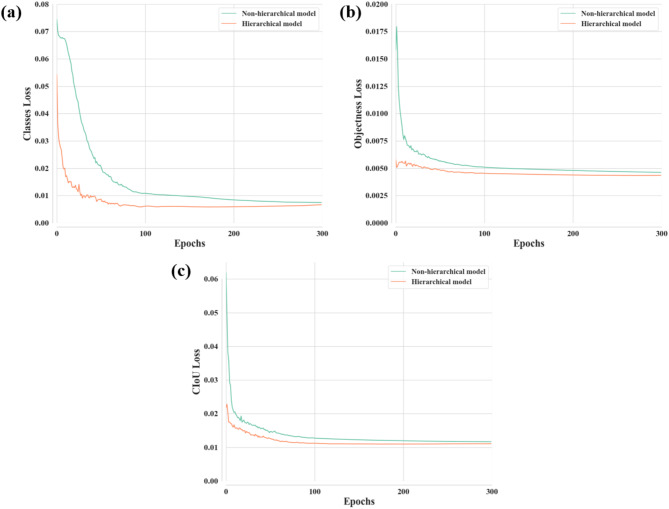
Comparison of loss analysis per epoch during training of the examined models. (a) Complete intersection over union (CIoU) loss, (b) Classes loss, and (c) Objectness loss.

**Fig. 4:**
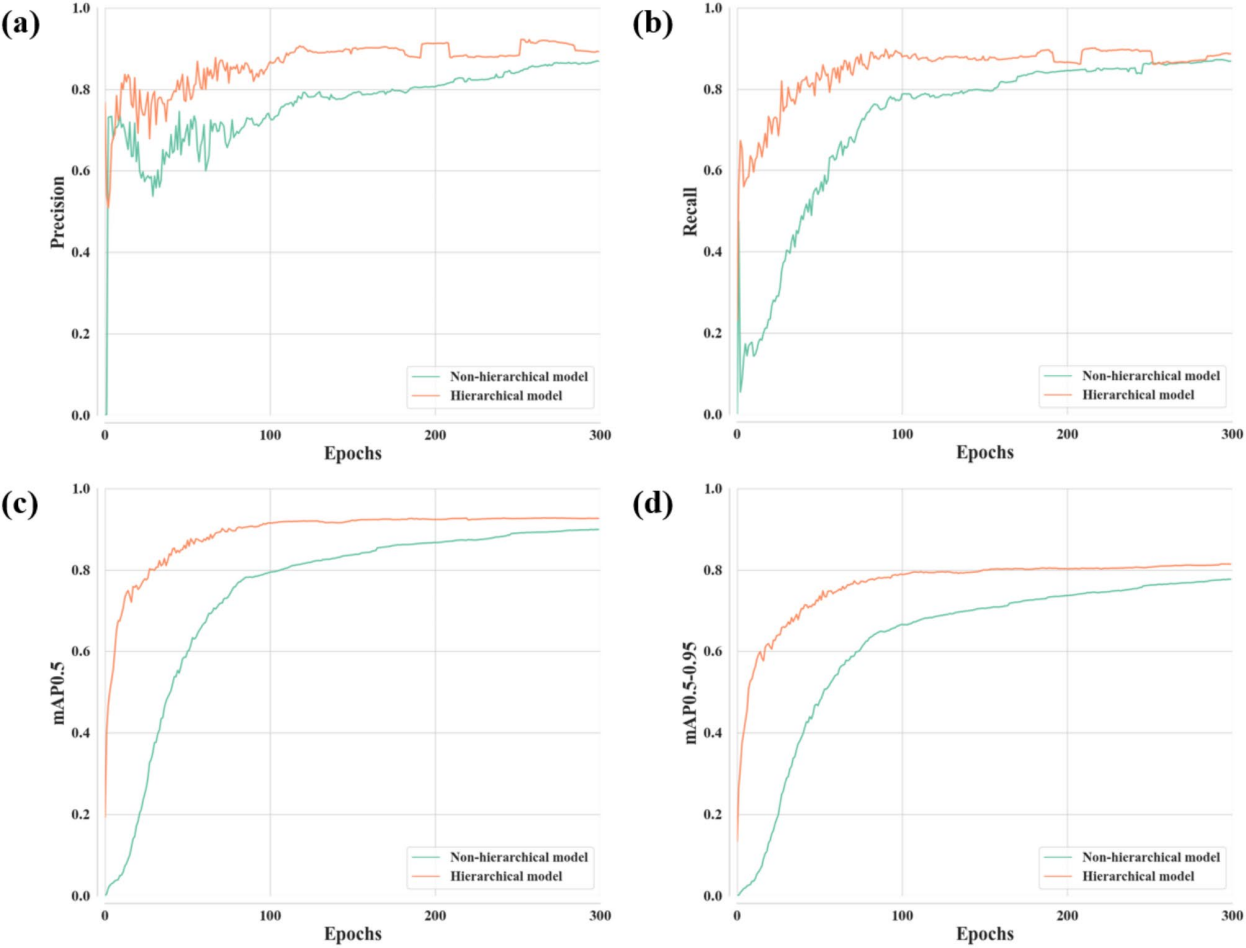
Comparison of evaluation results per epoch during training of the examined models. (a) Precision, (b) recall, (c) mean Average Precision when IoU is 0.5 (mAP0.5), and (d) mean Average Precision when IoU is 0.5–0.95 (mAP0.5–0.95).

Loss analysis per epoch during training of the non-hierarchical and hierarchical models is presented in Fig. 3. The loss values at the final epoch are presented in Table S6. The evaluation results per epoch during training of the class and subclass-level classification models are presented in Figures S1 and S2, respectively. In non-hierarchical and hierarchical models, all three losses, namely, CIoU, classes, and objectness, exhibited a decreasing trend, stabilizing eventually. The loss analysis indicated that neither the non-hierarchical nor the hierarchical model exhibited significant overfitting once the loss values plateaued and stopped decreasing further. The convergence of losses of the hierarchical model in the training process was better than that of the non-hierarchical model. At the final epoch, the losses of the hierarchical model were slightly smaller than those of the non-hierarchical model (Table S6). The CIoU loss was 0.01166 for the non-hierarchical model, and 0.01106 for the hierarchical model. The classes losses for non-hierarchical and hierarchical models were 0.00750 and 0.00666, respectively. Moreover, the objectness loss was 0.00463 for the non-hierarchical model and 0.00435 for the hierarchical model. The performance of the models during the training was evaluated at each epoch by calculating precision, recall, and mAP values (Fig. 4); the values of these variables at the final epoch are presented in Table S6. After the performance became stable and saturated, the hierarchical model showed higher values for all evaluation metrics than the non-hierarchical model. Specifically, two mAP values, reflecting precision and recall, were higher at every epoch for the hierarchical model than for the non-hierarchical model. Furthermore, at the final epoch, two mAP values of the hierarchical model were slightly higher than those of the non-hierarchical model (Table S6). The mAP0.5 was 0.899 for the non-hierarchical model and 0.926 for the hierarchical model. In addition, the mAP0.5–0.95 for non-hierarchical and hierarchical models were 0.777 and 0.814, respectively.

## *Comparison of performance after training completion*

**Fig. 5:**
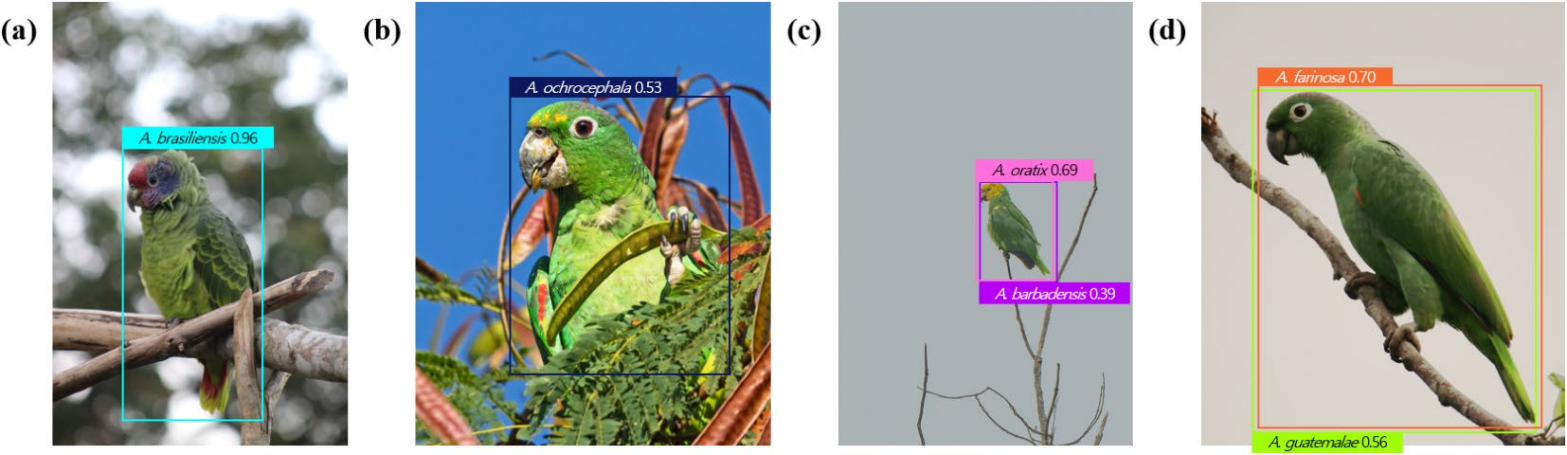
Representative images presenting four cases of model prediction results. Boxes represent prediction bounding boxes. The values within the boxes indicate confidence scores provided by the models, reflecting the probability of the predictions being accurate. (a) *Amazona brasiliensis*: A single prediction bounding box was predicted and correctly classified, (b) *Amazona farinosa*: A single prediction bounding box was predicted and incorrectly classified, (c) *Amazona oratrix*: Multiple prediction bounding box were predicted and correctly classified, and (d) *Amazona guatemalae*: Multiple prediction bounding box were predicted and incorrectly classified. Photo credit: (a) Hermann Moller, (b) Paulo Mascaretti, (c) Aitor, and (d) Gregg McClain.

The models produced four prediction outcomes after training completion, as presented in Fig. 5. A single bounding box could be predicted and classified correctly or incorrectly (Fig. 5a and b). In other cases, multiple bounding boxes could be predicted and classified correctly or incorrectly (Fig. 5c and d). For instances with multiple prediction bounding boxes, the classification result was determined by the bounding box with the highest confidence score suggested by the model, indicating the probability of the predictions being accurate. The overall evaluation results after completing the training of the examined models are presented in Table 1. Additionally, the evaluation results of each of the 35 Amazon parrot species are presented in Table S7. The evaluation results after finishing the training of the class and subclass-level classification models are presented in Tables S8 and S9, respectively. From the results of evaluating model performance, the hierarchical model showed higher values than the non-hierarchical model for all evaluation metrics (Table 1). For example, the precision values for the non-hierarchical and hierarchical models were 0.890 and 0.904, respectively. The lowest precision among 35 Amazon parrot species was 0.656 and 0.604 for *Amazona farinosa* in the non-hierarchical and hierarchical models, respectively. The highest precision of the examined species was 1.000 for *Amazona guildingii*, *Amazona imperialis*, and *Amazona lilacina* in the non-hierarchical model and 1.000 for *A. guildingii* and *A. imperialis*, in the hierarchical model. The recall value was 0.855 for the non-hierarchical model, while 0.899 for the hierarchical model. In both models, the recall of *A. farinosa* was the lowest: 0.480 in both non-hierarchical and hierarchical models. The recall of *A. albifrons* was the highest in both models: 0.986 for the non-hierarchical model and 0.990 for the hierarchical model. Meanwhile, the mAP0.5 was 0.908 for the non-hierarchical model and 0.944 for the hierarchical model. For the non-hierarchical model, AP0.5 ranged from 0.516 for *A. farinosa* to 0.993 for *Amazona aestiva*. For the hierarchical model, AP0.5 ranged from 0.579 for *A. farinosa* to 0.995 for *A. aestiva* and *A. imperialis*. Furthermore, mAP0.5–0.95 was 0.785 for the non-hierarchical model, compared with 0.835 for the hierarchical model. Similar to the AP0.5, AP0.5–0.95 was lowest for *A. farinosa*: 0.469 for the non-hierarchical model and 0.527 for the hierarchical model. In addition, the highest AP0.5–0.95 was 0.909 for *A. aestiva* in the non-hierarchical and 0.912 for *A. aestiva* and *A. viridigenalis* in the hierarchical models.

**Table 1:** Comparison of evaluation results after completing the training of the examined models.

Evaluation metrics	Non-hierarchical model	Hierarchical model
Precision	0.890	0.904
Recall	0.855	0.899
mAP0.5	0.908	0.944
mAP0.5-0.95	0.785	0.835

## Comparison of classification results after training completion

**Fig. 6:**
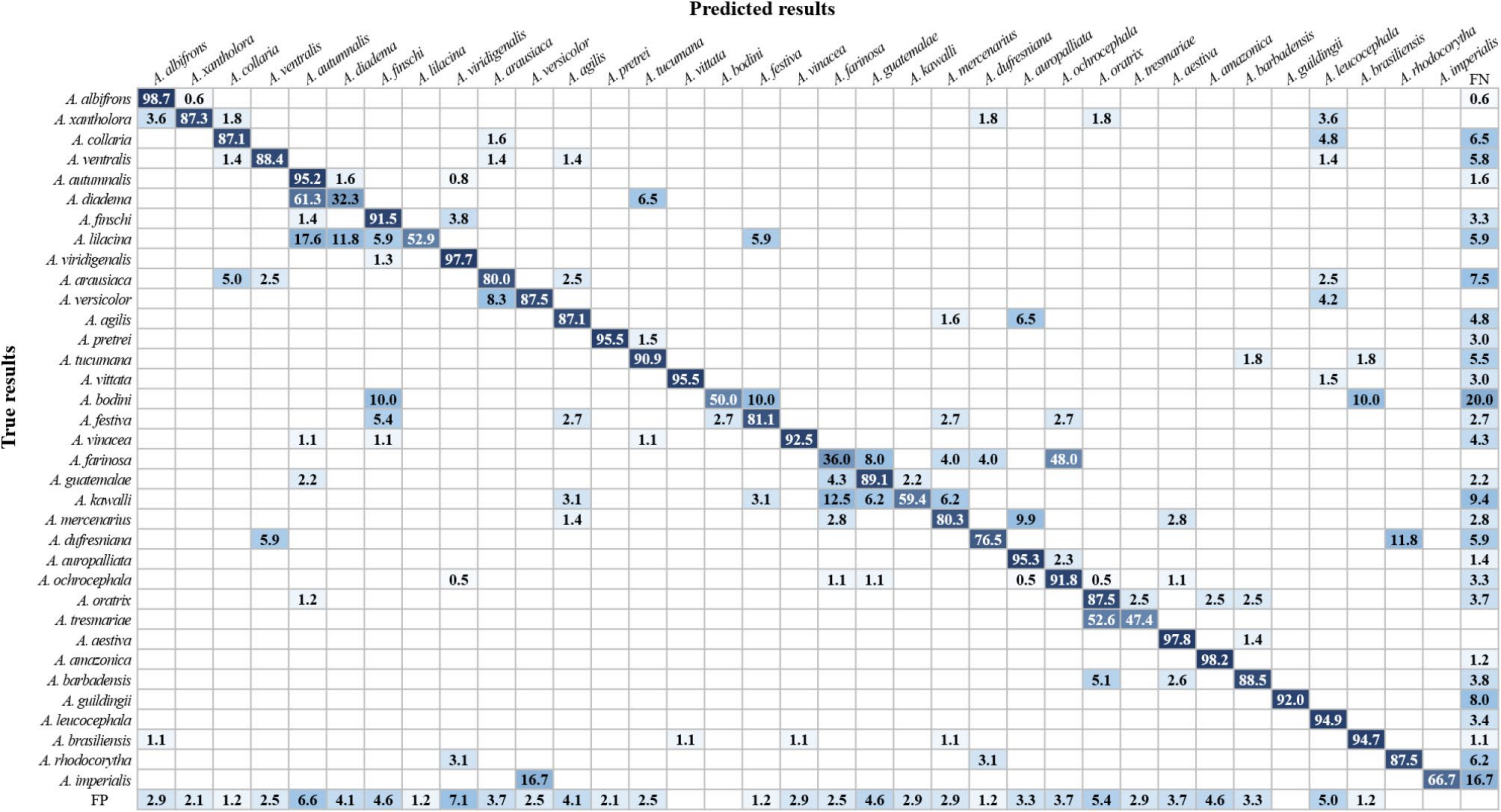
Confusion matrix of the non-hierarchical model representing the classification results of 35 Amazon parrot species. FP indicates background false positives, referring to the probability of mistakenly classifying Amazon parrots as the background. FN indicates background false negatives, referring to the probability of misclassifying background as Amazon parrots. The rows represent the actual species, while the columns represent the species predicted by the models. Prediction results are shown as percentage values. Diagonal values indicate correct predictions, while off-diagonal values represent incorrect predictions.

**Fig. 7:**
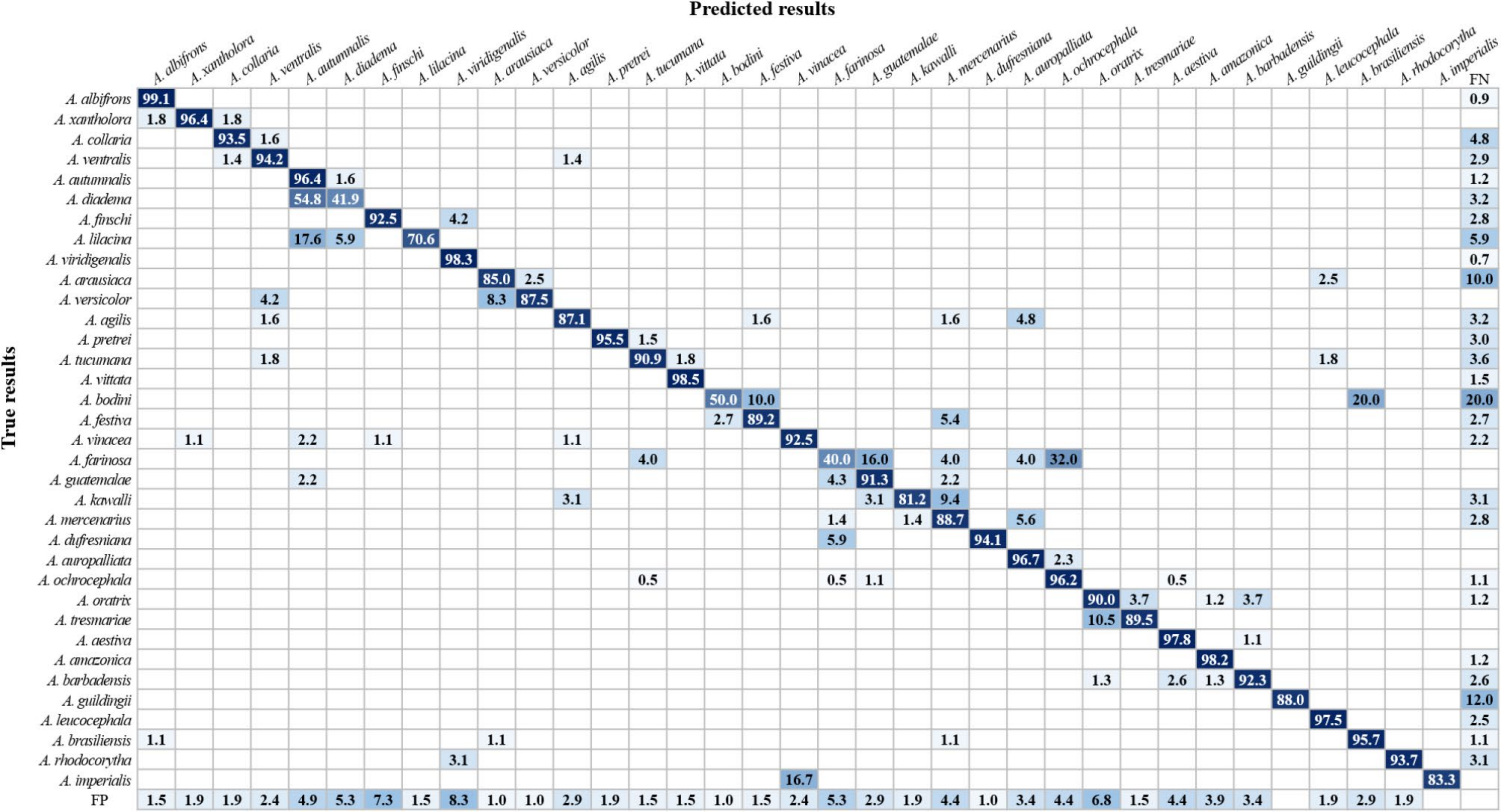
Confusion matrix of the hierarchical model representing the classification results of 35 Amazon parrot species. FP indicates background false positives, referring to the probability of mistakenly classifying Amazon parrots as the background. FN indicates background false negatives, referring to the probability of misclassifying background as Amazon parrots. The rows represent the actual species, while the columns represent the species predicted by the models. Prediction results are shown as percentage values. Diagonal values indicate correct predictions, while off-diagonal values represent incorrect predictions.

The classification results of 35 Amazon parrot species by the two examined models are presented as a confusion matrix (Figs. 6 and 7). For the non-hierarchical model, the correct classification rate was 82.1% on average, ranging from 32.3% for *Amazona diadema* to 98.7% for *A. albifrons*. Meanwhile, for the hierarchical model, the correct classification rate was 87.8% on average, ranging from 40.0% for *A. farinosa* to 99.1% for *A. albifrons*. In particular, the correct classification rate for *Amazona tresmariae* showed the greatest improvement between the two models, increasing from 47.4% with the non-hierarchical model to 89.5% with the hierarchical model. In addition, the correct classification rates for four species, namely, *Amazona dufresniana*, *A. imperialis*, *Amazona kawalli*, and *A. lilacina*, also increased by more than 15.0% in the hierarchical model compared with the rates in the non-hierarchical model. The misclassification rate among species within the same subclass tended to be higher than between species in different subclasses. In addition, among 16 subclasses, misclassification was more frequently detected in subclasses 3, 9, and 11 than in the others, for both non-hierarchical and hierarchical models (Figs. 6 and 7). In subclass 3, the misclassification rate for predicting *A. diadema* as *Amazona autumnalis* was the highest in the non-hierarchical model, at 61.3%, but decreased to 54.8% in the hierarchical model. Furthermore, the misclassification rates for predicting *A. lilacina* as *A. autumnalis*,* A. diadema* and *Amazona finschi* were 17.6%, 11.8%, and 5.9%, respectively, in the non-hierarchical model. In the hierarchical model, the misclassification rate for predicting *A. lilacina* as *A. diadema* or *A. finschi* decreased to 5.9% and 0.0%, respectively, while the misclassification rate for predicting *A. lilacina* as *A. autumnalis* remained unchanged from that with the non-hierarchical model. In subclass 9, the rate of misclassification of *A. kawalli* as *A. farinosa* was 12.5% in the non-hierarchical model, but it decreased to 0.0% in the hierarchical model. Additionally, the misclassification rate for predicting *A. farinosa* (subclass 9) as *Amazona ochrocephala* (subclass 11) was 48.0% in the non-hierarchical model, which decreased to 32.0% in the hierarchical model. This was the highest rate of misclassification between species from different subclasses in the two models. Finally, in subclass 11, the misclassification rate for predicting *A. tresmariae* as *Amazona oratrix* was 52.6% in the non-hierarchical model, which decreased to 10.5% in the hierarchical model. This represented the most substantial reduction in misclassification rate observed in the hierarchical model compared with that in the non-hierarchical model, indicating a marked improvement in classification accuracy. Moreover, the average misclassification rate that predicted objects as background false negative (FN) was decreased in the hierarchical model compared with that in the non-hierarchical model (Figs. 6 and 7). This misclassification refers to the likelihood of incorrectly identifying the background as an Amazon parrot, leading to the false detection of objects that are not actually present. This misclassification rate was 5.1% on average for the non-hierarchical model, ranging from 0.6% for *A. albifrons* to 20.0% for *Amazona bodini*, while it was 3.8% on average for the hierarchical model, ranging from 0.7% for *A. viridigenalis* to 20.0% for *A. bodini*. In particular, the background FN error rate of *A. imperialis* was the most improved in the hierarchical model compared with that in the non-hierarchical model. Owing to the improved background FN error rate, the rate of correct classification of this species increased from 66.7% with the non-hierarchical model to 83.3% with the hierarchical model.

## Discussion

Various studies have applied hierarchical classification for the classification of images of wildlife^28,34–36^. These studies constructed a hierarchy based on taxonomic assignment, involving classification being conducted following the different taxonomic levels, such as order, family, genus, and species levels. These proved that the hierarchical approach achieved improved classification accuracy. In the present study, hierarchical image classification using transfer learning was applied to enhance the performance of the deep learning model for classifying Amazon parrots. The hierarchy was constructed based on the morphological features. To the best of our knowledge, this study is the first to apply hierarchical classification based on morphological features to classify wildlife. Furthermore, although direct comparisons may not be appropriate due to differences in the dataset and model used in the studies, the hierarchical model developed in this study outperformed the model from the previous study, which was the first to apply a deep learning model for classifying Amazon parrots^2^. Specifically, the hierarchical model in this study achieved the mAP of 0.944, surpassing the mAP of 0.889 reported in the previous study. Moreover, the misclassification between morphologically similar species such as *A. vittata* and *A. tucumana* was 16.7% for the model in the previous study. However, only 0.0% of misclassification was observed for the hierarchical model developed in this study. These improvements highlight the effectiveness of the hierarchical classification approach in enhancing model performance for detecting and classifying Amazon parrots.

Evaluation results obtained during and after model training showed that the hierarchical model outperformed the non-hierarchical model in the detection and classification of Amazon parrots. Neither of the models was robust enough to achieve effective predictions, as indicated by the convergence of each loss function to less than 0.05 during training (Fig. 3)^56^. Moreover, the loss values for the hierarchical model were slightly lower than those of the non-hierarchical model (Fig. 3 and Table S6), suggesting that the predictions of the hierarchical model were closer to the true results^57^. According to the evaluation metrics, the hierarchical model demonstrated superior performance in detecting and classifying Amazon parrots compared with the non-hierarchical model, both during the training and after the training had been completed. Specifically, the mAP values for the hierarchical model were higher than those for the non-hierarchical model, not only during training (Fig. 4 and Table S6) but also after it had been completed (Table 1). This indicates that the hierarchical model improved in its capacity to accurately detect and classify the parrot species. Specifically, the hierarchical model showed enhanced classification accuracy for species that share morphological features. This improvement is crucial for monitoring wild populations and managing global trade, as accurate classification helps ensure effective conservation and regulation of trade in protected species.

The hierarchical model decreased the misclassification rate for predicting *A. diadema* as *A. autumnalis* compared with the non-hierarchical model. It also lowered the misclassification rates for predicting *A. lilacina* as *A. diadema* or *A. finschi*. Accurate classification among these four species is particularly important for conservation, owing to their different IUCN Red List statuses. While *A. autumnalis* and *A. diadema* are categorized as “Least Concern,” *A. finschi* and *A. lilacina* are listed as “Endangered” and “Critically Endangered,” respectively^43^. Furthermore, precise classification is essential because *A. autumnalis* is one of the top 10 most illegally traded parrots^58^. The hierarchical model also demonstrated a decreased misclassification rate of predicting *A. kawalli* as *A. farinosa* compared with the non-hierarchical model. These two species share overlapping distribution ranges and exhibit morphological similarities^46,47^. Therefore, accurate classification between these two species might be important for monitoring the wild population of *A. kawalli*, especially considering its trend of a declining population. Additionally, precise classification might be crucial for regulating the global trade of this species, as *A. kawalli* is listed in CITES Appendix II, meaning that its trade is controlled through permits^44^. The hierarchical model also reduced misclassification rates in predicting *A. farinosa* as *A. ochrocephala* compared with the non-hierarchical model. These two species were misclassified despite being grouped into different classes based on crown color. While *A. farinosa* typically has a greenish crown, some individuals exhibit variability with a yellowish crown^46,47^, which may have contributed to this misclassification. Accurate classification between these species is crucial for monitoring wild populations, as these are sympatric, ranging from Central to South America^45–47^. In particular, the precise classification of *A. farinosa* is crucial for managing global trade in wildlife, as this species is one of the top 10 most illegally traded parrots and is also listed in CITES Appendix II^58^. Finally, the hierarchical model reduced the misclassification rate for predicting *A. tresmariae* as *A. oratrix* compared with the non-hierarchical model. Accurate classification of these two species is particularly important for monitoring wild populations, as *A. oratrix* is categorized as “Endangered” in the IUCN Red List of Threatened Species^43^. Although *A. oratrix* is listed in CITES Appendix I, indicating that its trade is prohibited^44^, this species is one of those with the highest rates of illegal capture and smuggling^59^. Therefore, accurate classification of *A. oratrix* is crucial for regulating global trade.

Overall, the comparisons of model performance demonstrated that the hierarchical classification based on morphological features, using transfer learning, can enhance model performance in regard to detecting and classifying Amazon parrots, compared with the non-hierarchical model. In particular, this approach increased the classification accuracy between morphologically similar species compared with the non-hierarchical model. These improvements may be attributed to the hierarchical integration of morphological features, which enhances model training by guiding the structure and relationships among the features extracted by the model^28^. Although the hierarchical classification based on diagnostic morphological features improved the overall classification accuracy of Amazon parrots, classification accuracy for several species, including *A. bodini*, *A. diadema*, and *A. farinosa*, remained relatively lower than for other species (Fig. 7). This could be attributable to the unbalanced dataset, which typically results in poor model performance^2,9,60^. Although images were collected from three databases in this study while attempting to minimize data imbalance, the number of images still varied considerably, ranging from 24 for *A. imperialis* to 1,402 for *A. albifrons* (Table S1). This disparity may have contributed to the difficulty in achieving consistent classification accuracy across all species. Recently, generative adversarial network (GAN) models have been successfully applied to generate image data for wildlife, including rare species^26,61,62^. This approach has shown promise in augmenting datasets and improving the training of deep learning models for classifying wildlife. Therefore, in future work, GAN models should be applied to construct a more balanced dataset for Amazon parrots to improve classification performance, particularly for species for which relatively low classification accuracy was achieved, such as *A. bodini*, *A. diadema*, and *A. farinosa*. Moreover, deep learning models have recently undergone continuous developments, including the development of YOLOv9^63^ and YOLOv10^64^. Thus, in future research, these newly developed models should be used to classify Amazon parrots to further enhance model performance and improve classification accuracy.

In conclusion, this study employed hierarchical classification with transfer learning to enhance deep learning model performance in classifying Amazon parrots. The hierarchy for classifying 35 Amazon parrot species was constructed based on diagnostic morphological features. Knowledge from the class- and subclass-level classification model was transferred to the model classifying the 35 Amazon parrot species. The results of evaluations performed during and after model training showed that hierarchical classification improved the model performance in detecting and classifying Amazon parrots. Notably, this approach increased the accuracy of classifying species that share morphological features. To the best of our knowledge, this is the first study to apply the hierarchical classification based on morphological features to detect and classify Amazon parrots. The hierarchical model developed here has the potential to assist in monitoring wild populations and tracking the global trade of Amazon parrot species. Furthermore, ongoing advancements in deep learning models for detecting and classifying these parrots represent a crucial step toward establishing a reliable and accurate automated monitoring system.

## Electronic supplementary material

Below is the link to the electronic supplementary material.

Supplementary Material 1

## Data Availability

All data except images the authors do not have the right to share and trained models are available from the GitHub repository (https://github.com/kim2429/AmazonParrots).
