# Supplementary material for: Hierarchical image classification using transfer learning to improve deep learning model performance for amazon parrots
Source: Sci Rep. 2025 Jan 30;15:3790. doi: 10.1038/s41598-025-88103-3 (PMC11782500; doi:10.1038/s41598-025-88103-3)
Supplement: Supplementary file 1 — Supplementary Material 1 [file 41598_2025_88103_MOESM1_ESM.docx]

**Supplementary information**

**Hierarchical image classification using transfer learning to improve deep learning model performance for Amazon parrots**

Jung-Il Kim, Jong-Won Baek, and Chang-Bae Kim^*^

Biotechnology Major, Sangmyung University, Seoul 03016, South Korea

*****Email: evodevo@smu.ac.kr

**Table S1.** Number of images of the 35 Amazon parrot species collected from three databases.

| **Species** | **Taxonomic Species  Numbers (TSNs)** | **eBird** | **iNaturalist** | **Google** |
| --- | --- | --- | --- | --- |
| *Amazona albifrons* | 177785 | 1,402 | - | - |
| *Amazona xantholora* | 177808 | 166 | 33 | 30 |
| *Amazona collaria* | 177791 | 148 | 17 | 64 |
| *Amazona ventralis* | 177803 | 177 | 29 | 78 |
| *Amazona autumnalis* | 177788 | 1,030 | - | - |
| *Amazona diadema* | 714094 | 77 | 22 | 19 |
| *Amazona finschi* | 554925 | 455 | 283 | 88 |
| *Amazona lilacina* | 714637 | 3 | 3 | 57 |
| *Amazona viridigenalis* | 177806 | 1,291 | - | - |
| *Amazona arausiaca* | 177787 | 120 | 9 | 31 |
| *Amazona versicolor* | 177804 | 55 | 3 | 47 |
| *Amazona agilis* | 177784 | 206 | 6 | 54 |
| *Amazona pretrei* | 177801 | 97 | 6 | 87 |
| *Amazona tucumana* | 554924 | 136 | 16 | 60 |
| *Amazona vittata* | 177807 | 215 | 7 | 40 |
| *Amazona bodini* | 714639 | 1 | 3 | 23 |
| *Amazona festiva* | 177794 | 101 | 14 | 35 |
| *Amazona vinacea* | 177805 | 216 | 50 | 93 |
| *Amazona farinosa* | 177793 | 67 | 10 | 22 |
| *Amazona guatemalae* | 714656 | 134 | 57 | 18 |
| *Amazona kawalli* | 554928 | 59 | 3 | 11 |
| *Amazona mercenarius* | 1222622 | 217 | 8 | 40 |
| *Amazona dufresniana* | 177792 | 24 | 1 | 41 |
| *Amazona auropalliata* | 554927 | 790 | 100 | 59 |
| *Amazona ochrocephala* | 177800 | 552 | 183 | 69 |
| *Amazona oratrix* | 554926 | 175 | 126 | 59 |
| *Amazona tresmariae* | 714645 | 18 | 47 | 13 |
| *Amazona aestiva* | 177783 | 839 | 301 | 85 |
| *Amazona amazonica* | 177786 | 975 | 291 | 120 |
| *Amazona barbadensis* | 177789 | 236 | 18 | 79 |
| *Amazona guildingii* | 554929 | 35 | 3 | 71 |
| *Amazona leucocephala* | 177798 | 1,004 | - | - |
| *Amazona brasiliensis* | 177790 | 255 | 16 | 66 |
| *Amazona rhodocorytha* | 202266 | 52 | 13 | 71 |
| *Amazona imperialis* | 554930 | 3 | 1 | 20 |
| Total | | 11,331 | 1,679 | 1,650 |

**Table S2.** Information on the hierarchy based on diagnostic morphological features of 35 Amazon parrot species for training the pretrained model.

| **Class** | **Diagnostic morphological features** | | | **Subclass** | **Species** |
| --- | --- | --- | --- | --- | --- |
|  | **Primary** | **Secondary** | **Tertiary** |  |  |
| 1 | Blueish crown | White forehead | Red round eyes | 1 | *Amazona albifrons, Amazona xantholora* |
|  |  |  | White round eyes | 2 | *Amazona collaria, Amazona ventralis* |
|  |  | Red forehead | - | 3 | *Amazona autumnalis, Amazona diadema,  Amazona finschi, Amazona lilacina, Amazona viridigenalis* |
|  |  | Blue forehead | - | 4 | *Amazona arausiaca, Amazona versicolor* |
|  |  | Green forehead | - | 5 | *Amazona agilis* |
| 2 | Greenish crown | Red forehead | Green throat | 6 | *Amazona pretrei, Amazona tucumana, Amazona vittata* |
|  |  |  | Blue throat | 7 | *Amazona bodini, Amazona festiva* |
|  |  |  | Vinous-marron throat | 8 | *Amazona vinacea* |
|  |  | Green forehead | - | 9 | *Amazona farinosa, Amazona guatemalae,  Amazona kawalli, Amazona mercenarius* |
|  |  | Yellow forehead | - | 10 | *Amazona dufresniana* |
| 3 | Yellowish crown | Yellow coloration on head | - | 11 | *Amazona auropalliata, Amazona ochrocephala,  Amazona oratrix, Amazona tresmariae* |
|  |  | Blue coloration on head | - | 12 | *Amazona aestiva, Amazona amazonica,  Amazona barbadensis* |
| 4 | Whitish crown | Blueish cheeks | - | 13 | *Amazona guildingii* |
|  |  | Pinkish-red cheeks | - | 14 | *Amazona leucocephala* |
| 5 | Reddish crown | - | - | 15 | *Amazona brasiliensis, Amazona rhodocorytha* |
| 6 | Purplish crown | - | - | 16 | *Amazona imperialis* |

**Table S3.** Overview of the dataset of six classes grouped based on primary diagnostic morphological feature.

| **Class** | **Number of images** | **Training set** | **Validation set** | **Test set** |
| --- | --- | --- | --- | --- |
| 1 | 6,003 | 3,841 | 960 | 1,202 |
| 2 | 1,912 | 1,223 | 305 | 384 |
| 3 | 5,135 | 3,286 | 821 | 1,028 |
| 4 | 1,113 | 712 | 178 | 223 |
| 5 | 473 | 302 | 75 | 96 |
| 6 | 24 | 15 | 3 | 6 |
| Total | 1,4660 | 9,379 | 2,342 | 2,939 |

**Table S4.** Overview of the dataset of 16 subclasses grouped based on diagnostic morphological features.

| **Subclass** | **Number of images** | **Training set** | **Validation set** | **Test set** |
| --- | --- | --- | --- | --- |
| 1 | 1,631 | 1,043 | 260 | 328 |
| 2 | 513 | 328 | 82 | 103 |
| 3 | 3,328 | 2,129 | 532 | 667 |
| 4 | 265 | 169 | 42 | 54 |
| 5 | 266 | 170 | 42 | 54 |
| 6 | 664 | 424 | 106 | 134 |
| 7 | 177 | 113 | 28 | 36 |
| 8 | 359 | 229 | 57 | 73 |
| 9 | 646 | 413 | 103 | 130 |
| 10 | 66 | 42 | 10 | 14 |
| 11 | 2,191 | 1,402 | 350 | 439 |
| 12 | 2,944 | 1,884 | 471 | 589 |
| 13 | 109 | 69 | 17 | 23 |
| 14 | 1,004 | 642 | 160 | 202 |
| 15 | 473 | 302 | 75 | 96 |
| 16 | 24 | 15 | 3 | 6 |
| Total | 14,660 | 9,374 | 2,338 | 2,948 |

**Table S5.** Overview of the final dataset of 35 Amazon parrot species for training non-hierarchical and hierarchical models.

| **Species** | **Number of images** | **Training set** | **Validation set** | **Test set** |
| --- | --- | --- | --- | --- |
| *Amazona albifrons* | 1,402 | 897 | 224 | 281 |
| *Amazona xantholora* | 229 | 146 | 36 | 47 |
| *Amazona collaria* | 229 | 146 | 36 | 47 |
| *Amazona ventralis* | 284 | 181 | 45 | 58 |
| *Amazona autumnalis* | 1,030 | 659 | 164 | 207 |
| *Amazona diadema* | 118 | 75 | 18 | 25 |
| *Amazona finschi* | 826 | 528 | 132 | 166 |
| *Amazona lilacina* | 63 | 40 | 10 | 13 |
| *Amazona viridigenalis* | 1,291 | 826 | 206 | 259 |
| *Amazona arausiaca* | 160 | 102 | 25 | 33 |
| *Amazona versicolor* | 105 | 67 | 16 | 22 |
| *Amazona agilis* | 266 | 170 | 42 | 54 |
| *Amazona pretrei* | 190 | 121 | 30 | 39 |
| *Amazona tucumana* | 212 | 135 | 33 | 44 |
| *Amazona vittata* | 262 | 167 | 41 | 54 |
| *Amazona bodini* | 27 | 17 | 4 | 6 |
| *Amazona festiva* | 150 | 96 | 24 | 30 |
| *Amazona vinacea* | 359 | 229 | 57 | 73 |
| *Amazona farinosa* | 99 | 63 | 15 | 21 |
| *Amazona guatemalae* | 209 | 133 | 33 | 43 |
| *Amazona kawalli* | 73 | 46 | 11 | 16 |
| *Amazona mercenarius* | 265 | 169 | 42 | 54 |
| *Amazona dufresniana* | 66 | 42 | 10 | 14 |
| *Amazona auropalliata* | 949 | 607 | 151 | 191 |
| *Amazona ochrocephala* | 804 | 514 | 128 | 162 |
| *Amazona oratrix* | 360 | 230 | 57 | 73 |
| *Amazona tresmariae* | 78 | 49 | 12 | 17 |
| *Amazona aestiva* | 1,225 | 784 | 196 | 245 |
| *Amazona amazonica* | 1,386 | 887 | 221 | 278 |
| *Amazona barbadensis* | 333 | 213 | 53 | 67 |
| *Amazona guildingii* | 109 | 69 | 17 | 23 |
| *Amazona leucocephala* | 1,004 | 642 | 160 | 202 |
| *Amazona brasiliensis* | 337 | 215 | 53 | 69 |
| *Amazona rhodocorytha* | 136 | 87 | 21 | 28 |
| *Amazona imperialis* | 24 | 15 | 3 | 6 |
| Total | 14,660 | 9,367 | 2,326 | 2,967 |

**Table S6.** Comparison of training results at the final epoch of the examined models.

| **Metrics** | **Non-hierarchical model** | **Hierarchical model** |
| --- | --- | --- |
| CIoU loss | 0.01166 | 0.01106 |
| Classes loss | 0.00750 | 0.00666 |
| Objectness loss | 0.00463 | 0.00435 |
| Precision | 0.869 | 0.893 |
| Recall | 0.868 | 0.887 |
| mAP0.5 | 0.899 | 0.926 |
| mAP0.5-0.95 | 0.777 | 0.814 |

**Table S7.** Comparison of evaluation results after training completion of the models for classifying the 35 Amazon parrot species.

| **Species** | **Precision** | | | **Recall** | | **AP0.5** | | | **AP0.5-0.95** | |
| --- | --- | --- | --- | --- | --- | --- | --- | --- | --- | --- |
|  | **Non-hierarchical model** | **Hierarchical model** | **Non-hierarchical model** | | **Hierarchical model** | **Non-hierarchical model** | **Hierarchical model** | **Non-hierarchical model** | | **Hierarchical model** |
| *Amazona albifrons* | 0.971 | 0.977 | 0.984 | | 0.988 | 0.990 | 0.992 | 0.888 | | 0.903 |
| *Amazona xantholora* | 0.927 | 0.972 | 0.926 | | 0.982 | 0.955 | 0.990 | 0.801 | | 0.853 |
| *Amazona collaria* | 0.947 | 0.951 | 0.855 | | 0.944 | 0.926 | 0.950 | 0.814 | | 0.865 |
| *Amazona ventralis* | 0.952 | 0.902 | 0.864 | | 0.937 | 0.961 | 0.966 | 0.874 | | 0.900 |
| *Amazona autumnalis* | 0.889 | 0.901 | 0.968 | | 0.960 | 0.953 | 0.971 | 0.853 | | 0.880 |
| *Amazona diadema* | 0.731 | 0.694 | 0.526 | | 0.581 | 0.663 | 0.730 | 0.538 | | 0.654 |
| *Amazona finschi* | 0.940 | 0.942 | 0.939 | | 0.939 | 0.961 | 0.977 | 0.844 | | 0.869 |
| *Amazona lilacina* | 1.000 | 0.858 | 0.716 | | 0.709 | 0.833 | 0.902 | 0.697 | | 0.746 |
| *Amazona viridigenalis* | 0.924 | 0.945 | 0.980 | | 0.980 | 0.983 | 0.988 | 0.893 | | 0.912 |
| *Amazona arausiaca* | 0.848 | 0.903 | 0.836 | | 0.825 | 0.860 | 0.899 | 0.671 | | 0.708 |
| *Amazona versicolor* | 0.853 | 0.894 | 0.875 | | 0.917 | 0.949 | 0.946 | 0.744 | | 0.772 |
| *Amazona agilis* | 0.890 | 0.919 | 0.871 | | 0.918 | 0.939 | 0.977 | 0.798 | | 0.841 |
| *Amazona pretrei* | 0.925 | 0.958 | 0.938 | | 0.955 | 0.964 | 0.975 | 0.825 | | 0.879 |
| *Amazona tucumana* | 0.868 | 0.910 | 0.909 | | 0.919 | 0.938 | 0.966 | 0.854 | | 0.877 |
| *Amazona vittata* | 0.985 | 0.970 | 0.939 | | 0.965 | 0.979 | 0.983 | 0.873 | | 0.894 |
| *Amazona bodini* | 0.849 | 0.906 | 0.564 | | 0.700 | 0.714 | 0.902 | 0.555 | | 0.729 |
| *Amazona festiva* | 0.916 | 0.909 | 0.865 | | 0.919 | 0.928 | 0.970 | 0.827 | | 0.888 |
| *Amazona vinacea* | 0.934 | 0.934 | 0.903 | | 0.917 | 0.964 | 0.982 | 0.842 | | 0.885 |
| *Amazona farinosa* | 0.656 | 0.604 | 0.480 | | 0.480 | 0.516 | 0.579 | 0.469 | | 0.527 |
| *Amazona guatemalae* | 0.756 | 0.833 | 0.913 | | 0.957 | 0.918 | 0.928 | 0.833 | | 0.859 |
| *Amazona kawalli* | 0.902 | 0.904 | 0.750 | | 0.881 | 0.829 | 0.935 | 0.717 | | 0.828 |
| *Amazona mercenarius* | 0.882 | 0.808 | 0.844 | | 0.887 | 0.876 | 0.916 | 0.794 | | 0.836 |
| *Amazona dufresniana* | 0.746 | 0.867 | 0.824 | | 0.941 | 0.910 | 0.980 | 0.766 | | 0.873 |

**Table S7.** Continued

| **Species** | **Precision** | | **Recall** | | **AP0.5** | | **AP0.5-0.95** | |
| --- | --- | --- | --- | --- | --- | --- | --- | --- |
|  | **Non-hierarchical model** | **Hierarchical model** | **Non-hierarchical model** | **Hierarchical model** | **Non-hierarchical model** | **Hierarchical model** | **Non-hierarchical model** | **Hierarchical model** |
| *Amazona auropalliata* | 0.928 | 0.946 | 0.958 | 0.972 | 0.979 | 0.979 | 0.862 | 0.871 |
| *Amazona ochrocephala* | 0.899 | 0.916 | 0.925 | 0.973 | 0.965 | 0.972 | 0.874 | 0.891 |
| *Amazona oratrix* | 0.775 | 0.858 | 0.887 | 0.938 | 0.914 | 0.940 | 0.839 | 0.862 |
| *Amazona tresmariae* | 0.730 | 0.809 | 0.684 | 0.842 | 0.803 | 0.917 | 0.671 | 0.798 |
| *Amazona aestiva* | 0.960 | 0.979 | 0.986 | 0.990 | 0.993 | 0.995 | 0.909 | 0.912 |
| *Amazona amazonica* | 0.969 | 0.966 | 0.976 | 0.982 | 0.988 | 0.990 | 0.865 | 0.884 |
| *Amazona barbadensis* | 0.822 | 0.881 | 0.872 | 0.949 | 0.924 | 0.955 | 0.796 | 0.828 |
| *Amazona guildingii* | 1.000 | 1.000 | 0.868 | 0.863 | 0.977 | 0.965 | 0.661 | 0.753 |
| *Amazona leucocephala* | 0.930 | 0.972 | 0.941 | 0.962 | 0.967 | 0.984 | 0.820 | 0.845 |
| *Amazona brasiliensis* | 0.947 | 0.944 | 0.958 | 0.968 | 0.976 | 0.978 | 0.873 | 0.883 |
| *Amazona rhodocorytha* | 0.896 | 0.893 | 0.875 | 0.938 | 0.926 | 0.963 | 0.784 | 0.824 |
| *Amazona imperialis* | 1.000 | 1.000 | 0.736 | 0.876 | 0.872 | 0.995 | 0.761 | 0.905 |
| Average | 0.890 | 0.904 | 0.855 | 0.899 | 0.908 | 0.944 | 0.785 | 0.835 |

**Table S8.** Evaluation results after completing training of the class-level classification model.

| **Class** | **Precision** | **Recall** | **AP0.5** | **AP0.5-0.95** |
| --- | --- | --- | --- | --- |
| 1 | 0.940 | 0.966 | 0.984 | 0.881 |
| 2 | 0.911 | 0.888 | 0.951 | 0.842 |
| 3 | 0.956 | 0.965 | 0.980 | 0.876 |
| 4 | 0.937 | 0.897 | 0.943 | 0.782 |
| 5 | 0.924 | 0.880 | 0.948 | 0.856 |
| 6 | 1.000 | 0.000 | 0.535 | 0.488 |
| Average | 0.945 | 0.766 | 0.890 | 0.788 |

**Table S9.** Evaluation results after completing training of the subclass-level classification model.

| **Subclass** | **Precision** | **Recall** | **AP0.5** | **AP0.5-0.95** |
| --- | --- | --- | --- | --- |
| 1 | 0.992 | 0.978 | 0.992 | 0.910 |
| 2 | 0.983 | 0.975 | 0.990 | 0.879 |
| 3 | 0.958 | 0.969 | 0.989 | 0.893 |
| 4 | 0.974 | 0.912 | 0.974 | 0.787 |
| 5 | 0.905 | 0.921 | 0.967 | 0.840 |
| 6 | 0.942 | 0.913 | 0.951 | 0.842 |
| 7 | 0.945 | 0.912 | 0.974 | 0.871 |
| 8 | 0.961 | 0.925 | 0.972 | 0.865 |
| 9 | 0.928 | 0.936 | 0.964 | 0.874 |
| 10 | 0.794 | 0.824 | 0.903 | 0.824 |
| 11 | 0.946 | 0.963 | 0.970 | 0.870 |
| 12 | 0.969 | 0.977 | 0.988 | 0.882 |
| 13 | 0.988 | 0.923 | 0.964 | 0.728 |
| 14 | 0.974 | 0.957 | 0.986 | 0.854 |
| 15 | 0.969 | 0.932 | 0.975 | 0.895 |
| 16 | 1.000 | 0.595 | 0.855 | 0.798 |
| Average | 0.952 | 0.913 | 0.963 | 0.851 |

**
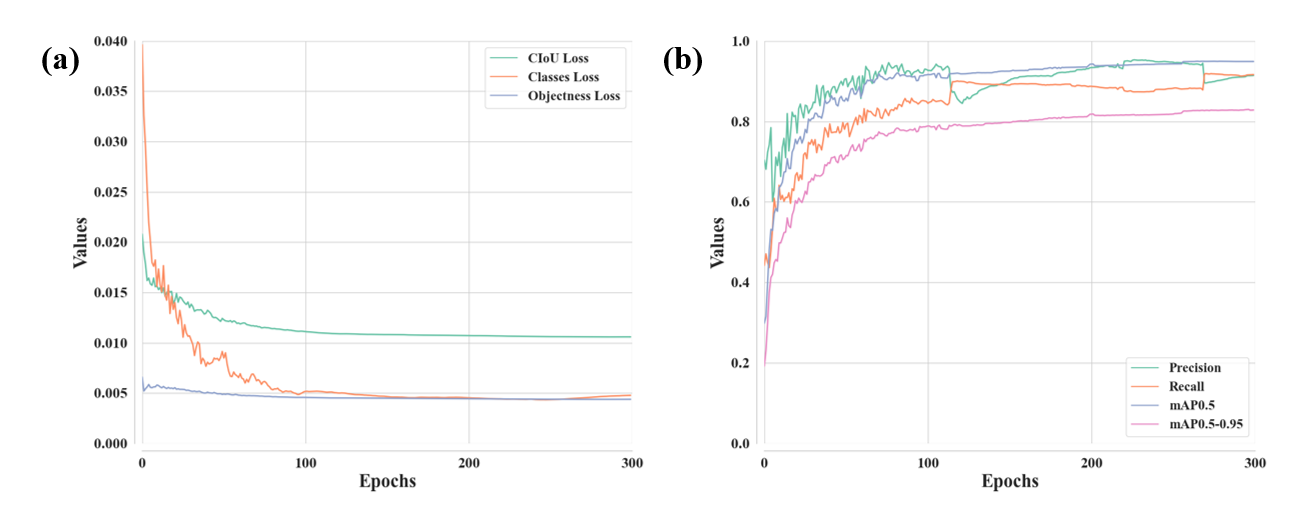
**

**Figure S1.** Evaluation results per epoch during training of the model classifying class level. **(**a) Loss functions, (b) Evaluation metrics.


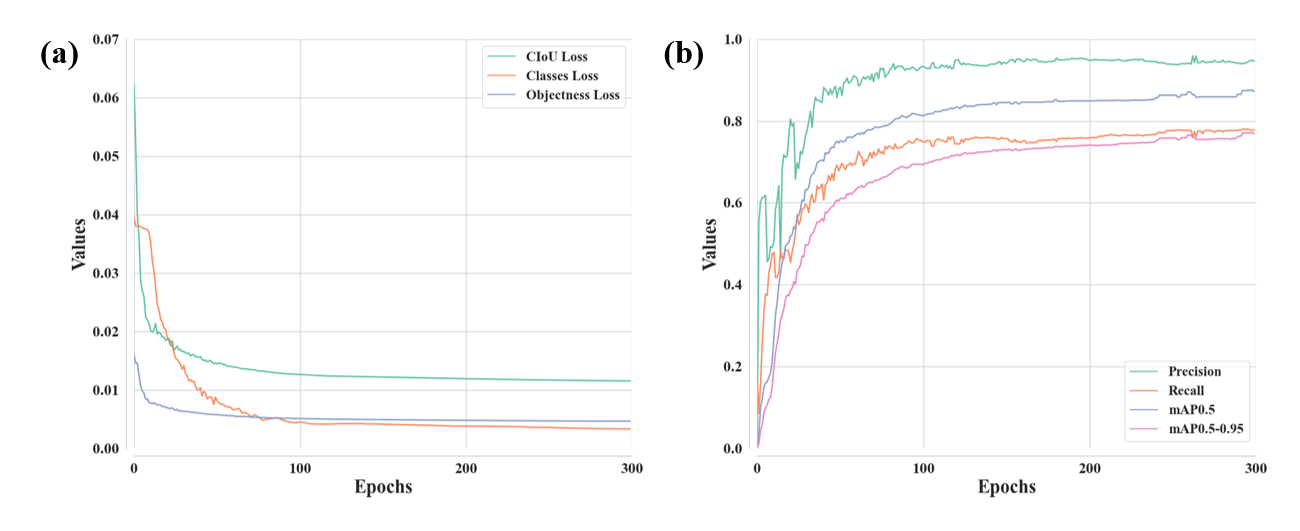


**Figure S2.** Evaluation results per epoch during training of the model classifying subclass level. (a) Loss functions, (b) Evaluation metrics.
